# Acupuncture and moxibustion in irritable bowel syndrome: a mechanistic exploration from heart rate variability to cardiac metabolism

**DOI:** 10.3389/fneur.2025.1716708

**Published:** 2025-12-15

**Authors:** Wenwei Wu, Yuchi Qiu, Fengxia Liang, Wei Lu, Yimeng Fu, Song Wu

**Affiliations:** 1 School of Acupuncture and Orthopedics, Hubei University of Chinese Medicine, Wuhan, China; 2 Hubei Shizhen Laboratory, Wuhan, China; 3 Hubei Collaborative Innovation Center of Acupuncture and Moxibustion, Wuhan, China; 4 Department of Acupuncture, Hubei Provincial Hospital of Traditional Chinese Medicine, Wuhan, China; 5 Administration Office, Hubei Maternal and Child Health Hospital, Wuhan, China; 6 The First Clinical College, Hubei University of Chinese Medicine, Wuhan, China

**Keywords:** irritable bowel syndrome, acupuncture, moxibustion, heart rate variability, autonomic nervous system

## Abstract

**Background:**

Patients with irritable bowel syndrome (IBS) exhibit abnormal heart rate variability (HRV). While acupuncture and moxibustion have shown therapeutic potential in IBS, the optimal acupoint selection strategy remains to be elucidated, and its effects on HRV and cardiac metabolomics in IBS models are incompletely understood. This study therefore aimed to compare the efficacy of two distinct acupoint protocols, BB (biao-ben protocol) and CG (conventional protocol), in modulating HRV and cardiac metabolomic profiles in a rat model of IBS.

**Methods:**

This study established an IBS rat model using chronic and acute stress (CAS). Two acupoint protocols were employed for the intervention of acupuncture combined with moxibustion: the BB group, which adhered to the fundamental Chinese medicine principle of “treating both the root and the branch” by selecting Neiguan (PC6), Guanyuan (CV4), and Zusanli (ST36); and the CG group, which utilized the most frequently used acupoints in clinical practice, namely Tianshu (ST25), Zusanli (ST36), and Shangjuxu (ST37). Visceral pain thresholds were measured by abdominal withdrawal reflexes (AWR). HRV was assessed using the BL-420F biofunctional experimental system. ELISA quantified atrial natriuretic peptide (ANP) and brain natriuretic peptide (BNP) levels in tissues and serum. Cardiac tissue metabolites analyzed through widely-targeted metabolomics and pathway enrichment performed via KEGG database.

**Result:**

Both BB and CG acupoint combinations significantly alleviated visceral pain thresholds in IBS rats. The BB protocol demonstrated superior efficacy in improving HRV parameters and regulating ANP and BNP levels in both serum and cardiac tissue. Metabolomic results revealed elevated concentrations of adenosine diphosphate (ADP), kynurenine, and kynurenic acid in the model group compared to the control, indicating disruptions in cardiac energy metabolism and inflammation-driven aberrations in tryptophan metabolism in IBS rats. The BB group exhibited downregulation of these metabolites.

**Conclusion:**

The BB acupoint combination may improve heart rate variability via autonomic nervous regulation, leading to the amelioration of cardiac energy metabolism and inflammation-driven tryptophan metabolic disorders.

## 1 Introduction

As a prevalent functional digestive condition, irritable bowel syndrome (IBS) presents with persistent visceral pain and variable stool consistency. According to the Rome III or IV criteria, the global prevalence of IBS is estimated to range from 3.8 to 9.2% (1). Dysfunction of the autonomic nervous system (ANS) has been identified as a key pathological mechanism in IBS (2, 3) and serves as a critical communication bridge in the bidirectional signaling of the brain-gut axis (4). When abnormal visceral sensory signals travel to the central nervous system via afferent pathways, they can disrupt the regulatory functions of brain regions (5, 6). On the other hand, anxiety and depression originating from the central nervous system may trigger sympathetic activation and vagal suppression, upsetting the balance of the autonomic nervous system (7). This imbalance then worsens intestinal motility and heightens visceral pain sensations (8, 9). Consequently, the functional status of the ANS has emerged as a crucial starting point for comprehending the complex clinical features of IBS.

Heart rate variability (HRV) is a key non-invasive indicator for assessment of ANS function, which accurately reflects the dynamic balance between sympathetic and parasympathetic nerve activities (2, 10). Numerous studies have shown that IBS patients generally have characteristic changes in HRV (11, 12). This heart-gut interaction originates from a common central integration center. Autonomic nerve fibers innervating the gastrointestinal tract and cardiovascular system converge in the nucleus tractus solitarius (NTS) of the medulla oblongata (13, 14). Abnormal sensory signals from the intestine are processed here (15, 16), and through “crosstalk” between different neuron groups within the NTS, they directly interfere with the autonomic nerve pathways that regulate cardiac rhythm (17). Therefore, abnormalities in HRV can be regarded as a quantifiable manifestation of intestinal dysfunction at the output of ANS (14).

In addition, atrial natriuretic peptide (ANP) and brain natriuretic peptide (BNP) secreted by the heart are considered key substances involved in stress response and emotional regulation (18, 19). However, current research on the changes in the levels of ANP and BNP in IBS and their association with disease symptoms is still very limited. To more comprehensively explore the overall metabolic response of the heart in IBS, we introduced cardiac metabolomics analysis.

Currently, the clinical management of IBS mainly relies on drug therapy, including spasmolytics, antidiarrheals, laxatives, and intestinal microbial modulators (20, 21). However, some drugs have a single target of action and can only treat individual symptoms such as abdominal pain, diarrhoea, or constipation, making it difficult to comprehensively address the complex and highly heterogeneous clinical symptom spectrum of IBS (22, 23). For patients with anxiety and depressed state, antidepressants are often syndicated (24), but such drugs may also cause side effects such as constipation, abdominal pain, and nausea (25, 26), which to a certain extent limit their clinical application.

Against this background, acupuncture and moxibustion have minimal adverse reactions and have been proven to significantly relieve symptoms in patients with IBS, including abdominal pain, abdominal distension, and increased frequency of bowel movements (27–29). Acupuncture may exert beneficial effects on IBS symptoms by regulating gastrointestinal motility, intestinal barrier function, visceral sensitivity, and brain-gut axis interaction (30). A major advantage of acupuncture and moxibustion lie in the flexibility of its acupoint combination, which can take both primary and secondary symptoms into account. In response to the commonly observed reduction in HRV among patients with irritable bowel syndrome, we proposed the “Biao-Ben” acupoint combination, selecting Neiguan (PC 6), Zusanli (ST 36), and Guanyuan (CV 4) for the intervention. Derived from the traditional Chinese medicine theory of “treating both the root and the branch,” this approach aims to integrate acupoints known to regulate gastrointestinal function to alleviate symptoms, with those that modulate the autonomic nervous system to address the underlying cause.

Multiple studies have demonstrated that acupoints such as PC6, ST36, and CV4 can improve HRV. Additionally, moxibustion at CV4 and ST36 has been shown to enhance autonomic nervous system function in patients with chronic fatigue syndrome, with sustained long-term effects (31). ST36 has been found to ameliorate visceral hypersensitivity and anxiety-like behaviors by modulating functional connectivity between the anterior cingulate cortex and the anterior insula, suggesting a potential central mechanism for enhancing vagal activity (32). Clinical studies indicate that acupuncture at PC6 can increase vagal activity in subjects (33, 34), potentially by activating the nucleus ambiguus to enhance vagal output while suppressing sympathetic outflow from the paraventricular nucleus and the rostral ventrolateral medulla, thereby restoring autonomic nervous system balance (35). Previous studies by our research group have attested that this acupoint combination can improve intestinal power supply by up-regulating the expression of key intestinal mucosal metabolic enzymes (Bpnt1) and mitochondrial ATP synthase subunit (ATP5A) (36). It can also downregulate the plasma Norepinephrine (NE) level (37), suggesting that it may regulate the sympathetic-parasympathetic balance. To evaluate the efficacy of the BB protocol, this study established a control group using a conventional protocol (CG). The acupoints for CG—Zusanli (ST36), Tianshu (ST25), and Shangjuxu (ST37)—were selected based on their status as the three most frequently utilized points in previous data mining studies on IBS treatment (38). This study aims to elucidate the mechanisms underlying the differential therapeutic effects produced by distinct acupuncture and moxibustion point selection protocols.

## 2 Materials and methods

### 2.1 Experimental animals

A total of 24 male Sprague–Dawley rats (3 months old, weighing 300 ± 20 g) were obtained from China Three Gorges University (Experimental animal production license number: SCXK2017-0012). All rats were housed under controlled conditions with a temperature of 22 ± 2 °C, humidity of 50 ± 10%, and a 12-h light/dark cycle.

### 2.2 Creation of the IBS model

The IBS rat model was established using the chronic and acute stress (CAS) method (39). Seven distinct stressors were applied, including 24-h water deprivation, 24-h food deprivation, 1-min painful tail pinching, 5-min exposure to 45 °C water, 3-min swimming in 4 °C water, 12-h day/night inversion, and 45-min horizontal vibration (120 rpm). Each day for 3 weeks (21 days total), two stressors were randomly selected by lottery for model induction. To avoid repetition, no stressor was applied on two consecutive days; if duplication occurred, a new stressor was selected. After a 1-week rest period, acute restraint stress was applied on the 28th day by wrapping the rats’ shoulders, upper limbs, forelimbs, and thoracic region for 1 h. Visceral pain thresholds were measured immediately after the acute stress. Successful model establishment was confirmed if the visceral pain threshold in the IBS group was significantly higher than that in the control group (*p* < 0.05).

### 2.3 Experimental design and groups

Rats without model establishment were assigned to the control group (CT), while those that underwent model establishment were randomly divided into the irritable bowel syndrome group (IBS), the Biao-Ben acupoint combination group (BB), and the conventional acupoint combination group (CG). There were six rats in each group (n = 6).

### 2.4 Intervention methods

In the BB group, three acupoints were selected: Neiguan (PC 6, located on the inner side of the forelimb, 3 mm proximal to the carpal joint between the radial and ulnar bones), Zusanli (ST 36, located on the posterolateral side of the knee joint, 3 mm below the fibular tuberosity), and Guanyuan (CV 4, located 25 mm below the umbilicus). In the CG group, three acupoints were selected: Zusanli (ST 36), Shangjuxu (ST 37, located in the depression between the tibialis anterior and extensor digitorum longus muscles, approximately 5–7 mm below the knee joint), and Tianshu (ST 25, located approximately 5 mm lateral to the umbilicus).

The rats received daily acupuncture-moxibustion treatment followed by CAS modeling on the same day. This treatment protocol was continued for 4 weeks (once daily). During the procedure, rats in the BB and CG groups were gently restrained using homemade cloth bags to minimize stress. In the BB group, sterile acupuncture needles (0.30 mm × 13 mm, Hua Tuo Brand, Suzhou Medical Supplies Factory Limited, Suzhou, China) were inserted into the CV4 acupoint and bilateral ST36 and PC6 acupoints. In the CG group, needles were inserted into bilateral ST36, ST37, and ST25 acupoints. The needles were manipulated using a balanced tonifying and reducing technique at a frequency of 120 twists per minute, with the needle handle rotated every 4 min for 1 min. Homemade moxa sticks (12 cm in length, 7 mm in diameter) were suspended 2–3 cm above the acupoints using a stand, and moxibustion was performed. Care was taken to ensure that the rats remained calm throughout the treatment.

### 2.5 Abdominal withdrawal reflex test

The AWR test was conducted before modeling and on day 28. Rats were fasted overnight with free access to water prior to testing. During the procedure, one experimenter gently restrained the rat and exposed the anus, while another inserted a custom-made balloon catheter (marked at 4.5 cm) lubricated with liquid paraffin into the anus using a cotton swab. The catheter was secured at the base of the tail with medical tape. After placing the rat on the operating table and allowing it to acclimate, one experimenter gradually inflated the balloon using a homemade sphygmomanometer, while the other recorded the pressure at which the rat exhibited abdominal lifting or back arching. Each rat was tested three times at 5-min intervals, and the average value was calculated.

### 2.6 Heart rate variability measurement

Heart rate variability was measured using the BL-420F biofunctional experimental system. Rats were anesthetized via isoflurane inhalation and connected to four subcutaneous standard limb leads. After the ECG waveforms stabilized, changes in the surface ECG and heart rate were recorded through standard type II limb leads. The following HRV parameters were analyzed: the standard deviation of normal sinus RR intervals (SDNN), the root mean square of successive differences between normal RR intervals (RMSSD), the mean R-R interval (RR Mean), total power (TP), very low frequency power (VLF), low frequency power (LF), high frequency power (HF), and the ratio of low frequency to high frequency power (LF/HF).

### 2.7 Enzyme linked immunosorbent assay assessment

At the end of the 28-day treatment period, rats were fasted overnight without water. On day 29, rats were anesthetized with 2% sodium pentobarbital (0.2 mL/100 g) and immobilized on a surgical plate. The abdominal cavity was opened, and blood was collected from the abdominal aorta. The rats were then immediately euthanized by cervical dislocation under deep anesthesia to ensure rapid loss of consciousness. Blood samples were allowed to clot for 1 h at room temperature, followed by centrifugation at 4 °C and 3,000 rpm for 10 min. The supernatant was transferred into labeled 1 mL centrifuge tubes. After blood collection, heart tissues were excised, rinsed with PBS, gently blotted dry with filter paper, flash-frozen in liquid nitrogen, and stored at −80 °C for further analysis.

For tissue processing, cardiac tissues were placed in pre-chilled 1.5 mL centrifuge tubes. Ice-cold RIPA lysis buffer (containing 1% protease inhibitor cocktail) was added at a volume of 1 mL, and tissues were homogenized on ice using a tissue homogenizer until fully disrupted. The homogenates were incubated at 4 °C for 30 min to ensure complete lysis, followed by centrifugation at 12,000 rpm for 15 min at 4 °C. The supernatant was carefully collected, avoiding the pellet, and transferred to new pre-chilled centrifuge tubes. Levels of ANP and BNP in both serum and heart tissue were quantified using commercially available enzyme linked immunosorbent assay (ELISA) kits (HyCell Biotechnology, Wuhan, Hubei, China) according to the manufacturer’s protocols.

### 2.8 Preparation of cardiac samples and metabolite extraction

Before analysis, samples were thawed on ice after removal from the −80 °C freezer. After thawing, residual blood on the sample surface was removed with filter paper. A portion of the sample was excised using a scalpel, transferred to a pre-cleaned Eppendorf tube with forceps, and weighed to achieve a consistent mass of 50 ± 2 mg, with the weight recorded accurately. A steel ball was added to the weighed sample, and the mixture was homogenized four times at 30 Hz for 30 s each. Subsequently, 1 mL of a 70% methanol solution containing internal standards was added dropwise to the homogenized sample in the centrifuge tube. The tube was shaken for 5 min and then left on ice for 15 min. The sample was then centrifuged at 12,000 rpm for 10 min at 4 °C. After centrifugation, 400 μL of the supernatant was aspirated and transferred to a corresponding EP tube, which was stored overnight in a −20 °C freezer. The next day, the sample was centrifuged again at 12,000 rpm for 3 min at 4 °C. Finally, 200 μL of the supernatant was carefully transferred to the corresponding sample vial liner for further analysis.

### 2.9 Widely-targeted metabolomics

Firstly, the sample was scanned with secondary spectrum by high-resolution Quadrupole-Time of Flight (TOF). The MRM ion pair information was extracted. After integrating with broad-target libraries, such as the Metware self-built database, and public databases including HMDB,^1^ MoNA,^2^ MassBank,^3^ METLIN,^4^ NIST,^5^ and MetDNA,^6^ the substances in the high-resolution samples were qualitatively analyzed. Subsequently, the qualitative substance ion pairs and RT (retention time) were transferred to the LC-MS for data acquisition. Peak areas were obtained to construct a database of the specificity of the samples. Finally, MRM accurate detection of the library substances was carried out using triple quadrupole linear ion trap mass (QTRAP).

### 2.10 Data processing and statistical analysis

PCA analysis was performed using the software R. Partial least squares discrimination analysis (OPLS-DA) was performed using the software Metaboanalyst R (R). Metabolites were analyzed and enriched metabolic pathways were found through KEGG (Kyoto Encyclopedia of Genes and Genomes) database.^7^

GraphPad Prism 8.3.0 (GraphPad, New York, United States) was utilized for data analysis. Normally distributed measurement data are expressed as mean ± standard deviation (mean ± SD). Comparisons between two groups were conducted using the independent-samples *t*-test, whereas comparisons among multiple groups were performed through one-way ANOVA (analysis of variance). *Post hoc* pairwise comparisons were carried out using Tukey’s method following ANOVA. Statistical significance was established at *p* < 0.05, with a more stringent significance threshold set at *p* < 0.01.

## 3 Results

### 3.1 Visceral pain thresholds of rats

**Figure 1:**
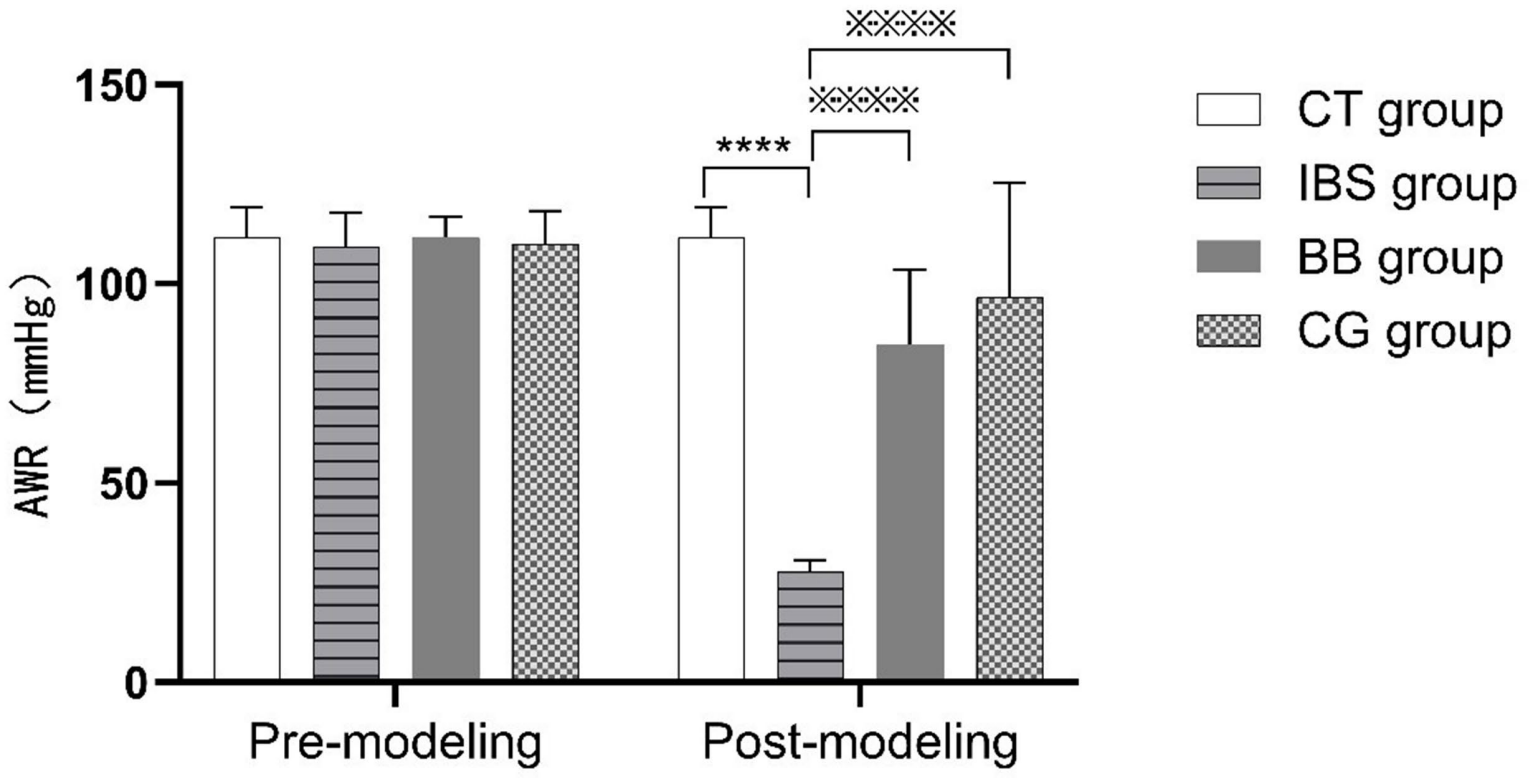
Comparison of visceral pain thresholds in rats before (pre-modeling) and after (post-modeling) model establishment. (^****^p < 0.01 compared to the CT group; ^※※※※^*p* < 0.01 compared to the IBS group).

The visceral pain thresholds in rats were measured using AWR. The results are shown in Figure 1. After modeling, the visceral pain threshold in the IBS group was significantly lower than that in the CT group, indicating successful model establishment. In contrast, the thresholds in both the BB and CG groups were significantly higher than in the IBS group, suggesting that acupuncture treatment effectively improved visceral hypersensitivity.

### 3.2 Heart rate variability in rats

**Figure 2:**
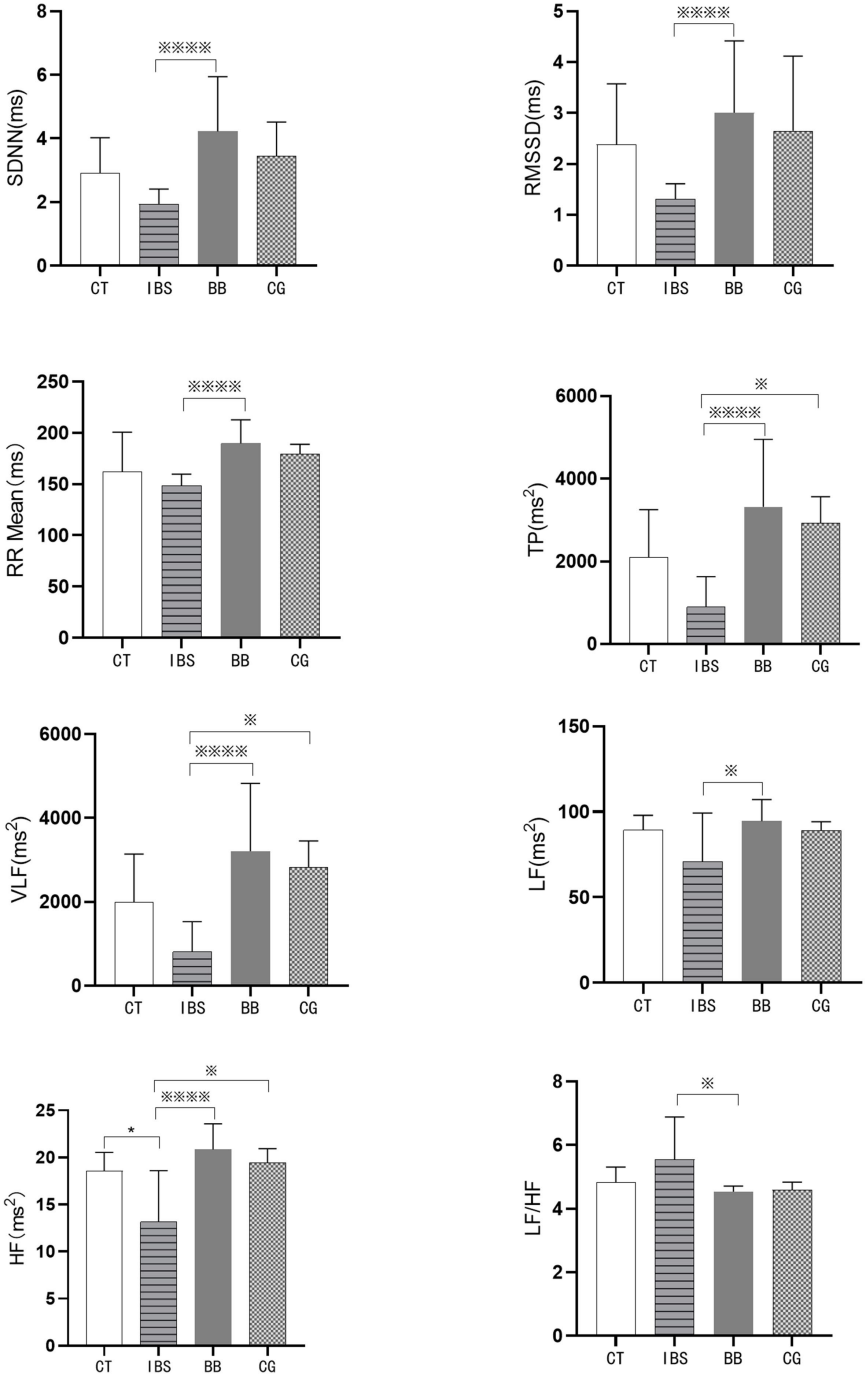
Comparison of the main HRV indices in rats. (^*^p < 0.05 compared to the CT group; ^※※※※^*p* < 0.01 and ^※^*p* < 0.05 compared to the IBS group).

Performed statistical analysis on HRV-related indicators, including the standard deviation of normal-to-normal intervals (SDNN), the root mean square of successive differences (RMSSD), the mean of RR intervals (RR Mean), total power (TP), very low frequency (VLF), low frequency (LF), high frequency (HF), and the ratio of low frequency to high frequency (LF/HF). The results are shown in Figure 2.

Statistically significant differences were observed in the HF values between the IBS group and the CT group (*p* < 0.05). Comparison of the BB group with the IBS group revealed significant differences in multiple HRV parameters, including SDNN, RMSSD, RR Mean, TP, VLF, LF, HF, and LF/HF (*p* < 0.01, *p* < 0.05). Compared with the IBS group, the CG group showed statistically significant differences in TP, VLF, and HF values (*p* < 0.05). No significant differences were observed in SDNN, RMSSD, RR Mean, TP, VLF, LF, HF, or LF/HF between the CG group and the BB group (*p* > 0.05).

### 3.3 ELISA analysis of ANP and BNP in serum and cardiac tissue

**Figure 3:**
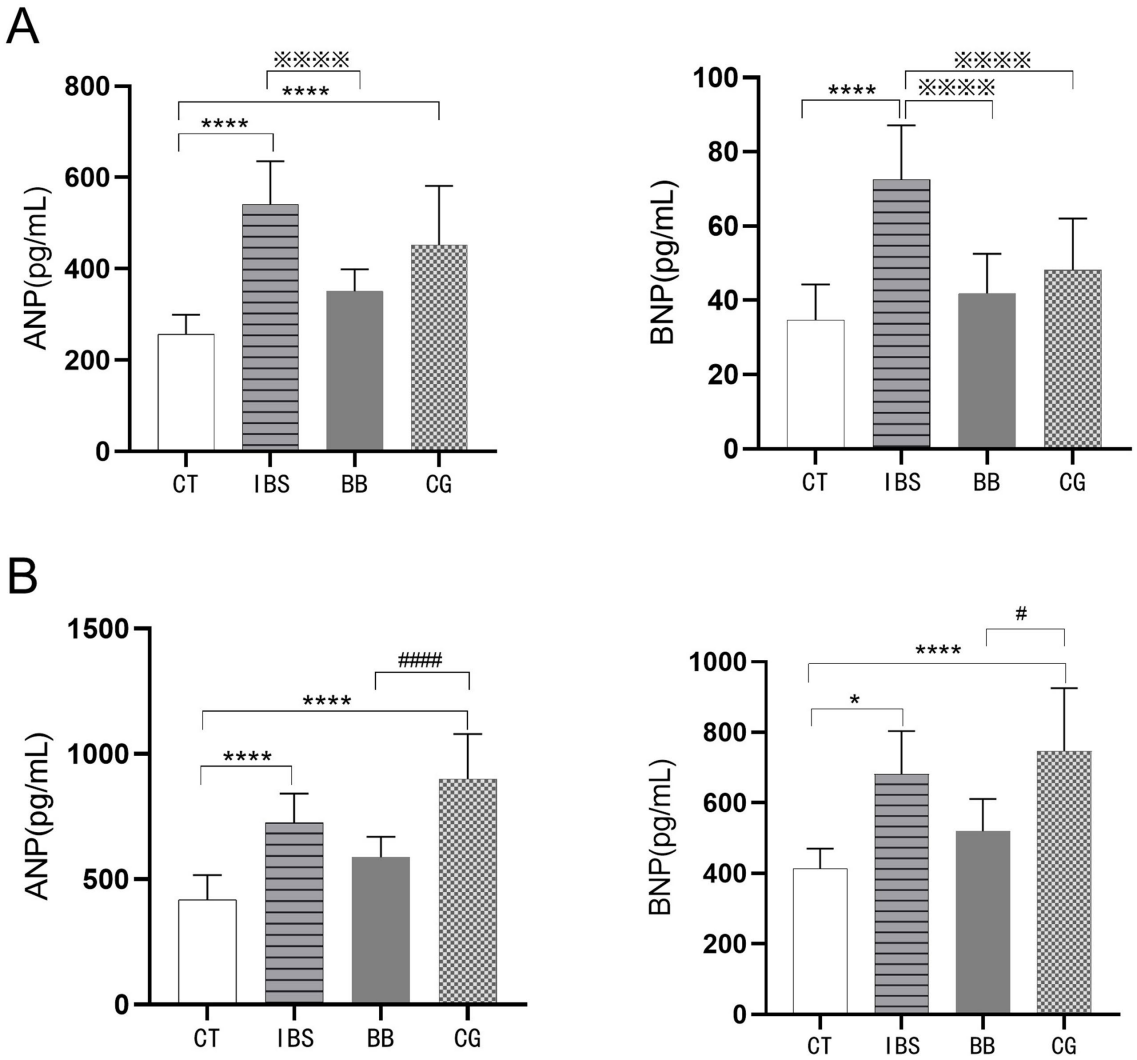
Comparison of ANP and BNP levels. **(A)** The contents of ANP and BNP in serum. **(B)** The contents of ANP and BNP in the heart. (^****^p < 0.01 and ^*^p < 0.05 compared to the control group; ^※※※※^*p* < 0.01 compared to the IBS group; ^####^*p* < 0.01 and ^#^*p* < 0.05 compared to the BB group).

Compared to the CT group, the IBS group showed significantly increased levels of ANP and BNP in both serum and cardiac tissue (Figure 3). Regarding serum indicators (Figure 3A), the BB group exhibited significant reductions in both ANP and BNP levels compared to the IBS group, while the CG group only showed a significant decrease in BNP with no statistically significant change in ANP. In cardiac tissue analysis (Figure 3B), the BB group displayed non-significant reductions in ANP and BNP levels compared to the IBS group, whereas the CG group showed non-significant increases. Notably, significant differences in cardiac ANP and BNP levels were observed between the BB and CG groups.

### 3.4 Principal component analysis analysis of overall samples

**Figure 4:**
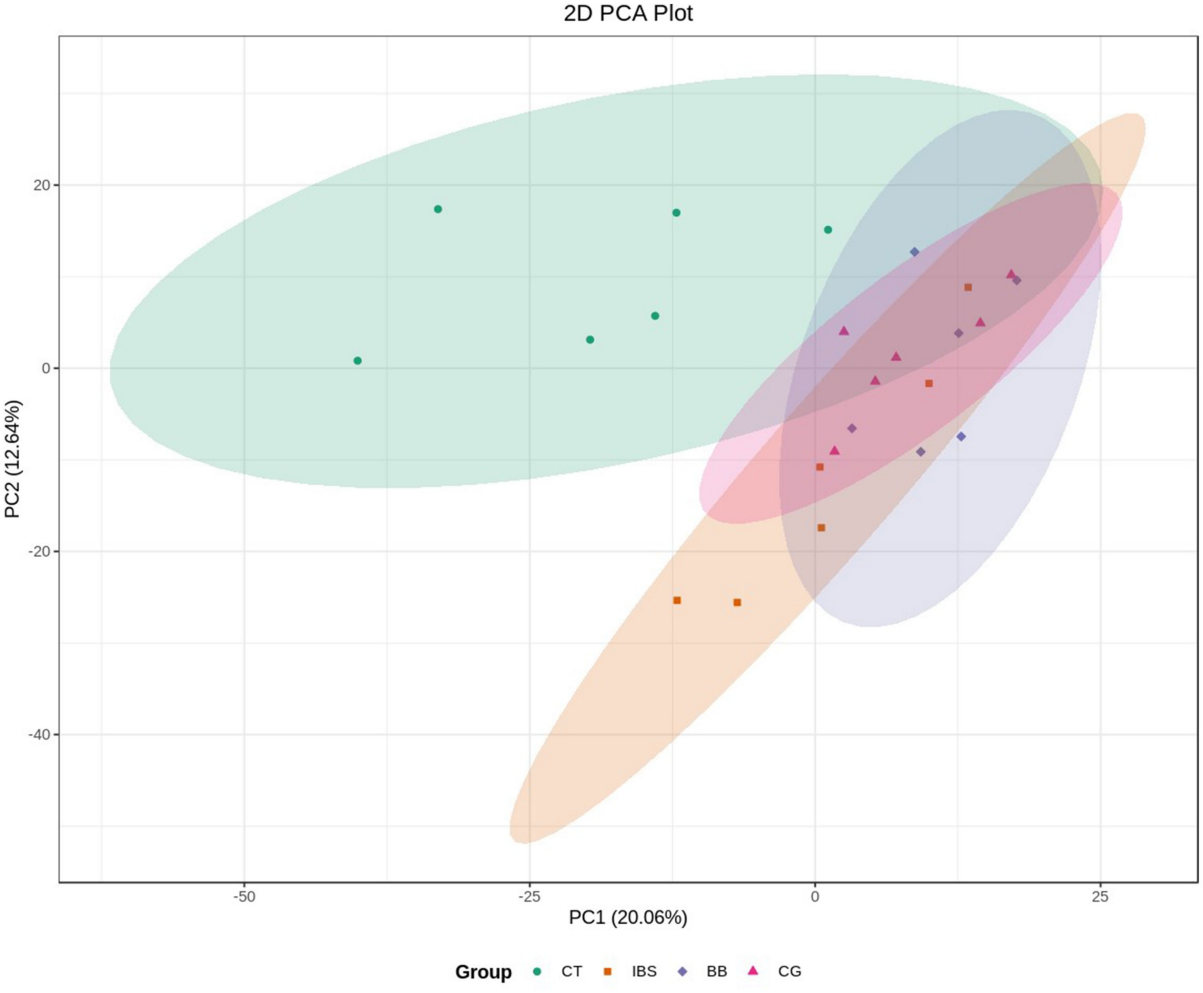
PCA score plot for overall samples. Horizontal axis: PC1; vertical axis: PC2. Percentages represent the contribution of each principal component to the total variance among samples. Each point in the plot corresponds to one sample, and samples from the same group are color-coded.

Principal component analysis (PCA) was performed on the metabolic profiles of all samples (Figure 4). This analysis aimed to identify overall metabolic differences between the groups and to assess the variability among samples within each group. The results showed that the first principal component (PC1) accounted for 20.6% of the variance in the original data, while the second principal component (PC2) explained 12.64%. Separation was observed along both PC1 and PC2. However, due to the inclusion of multiple test groups, the figure did not demonstrate clear categorization. Further PCA analysis was conducted to compare samples across groups.

### 3.5 PCA of grouped samples

**Figure 5:**
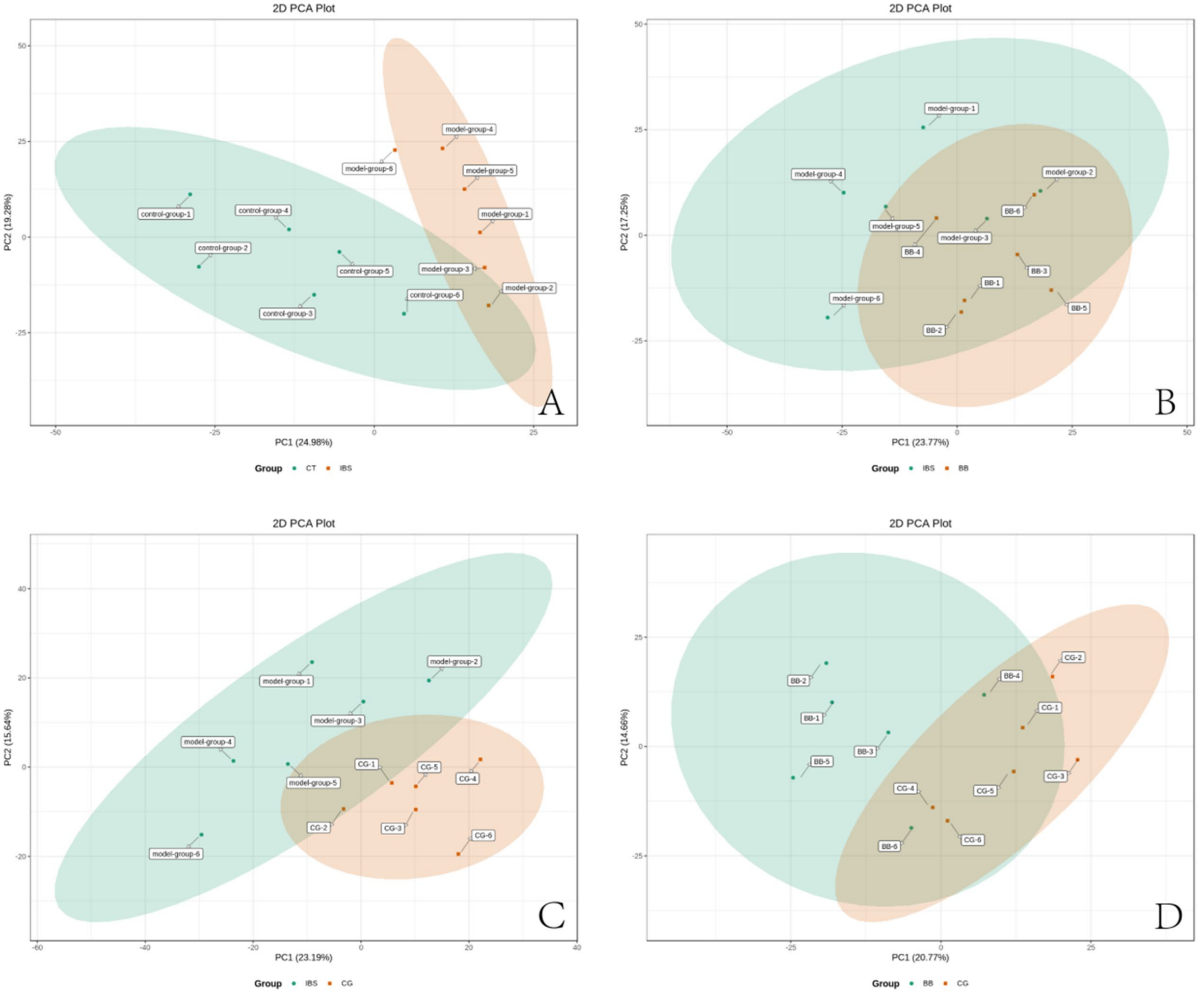
PCA score plot for four groups of rats. **(A)** CT group compared with IBS group. **(B)** IBS group compared with BB group. **(C)** IBS group compared with CG group. **(D)** BB group compared with CG group.

PCA was performed to compare the metabolic profiles of the four sample groups (Figure 5). Clear clustering was observed within each group, with only a few outliers detected. These results indicated that the data from the four groups were stable. The IBS group was separated from the CT group based on the PC1 (Figure 5A), but no significant differences were observed between the IBS group and the BB group (Figure 5B), the IBS group and the CG group (Figure 5C), or the BB group and the CG group (Figure 5D).

### 3.6 OPLS-DA analysis

**Figure 6:**
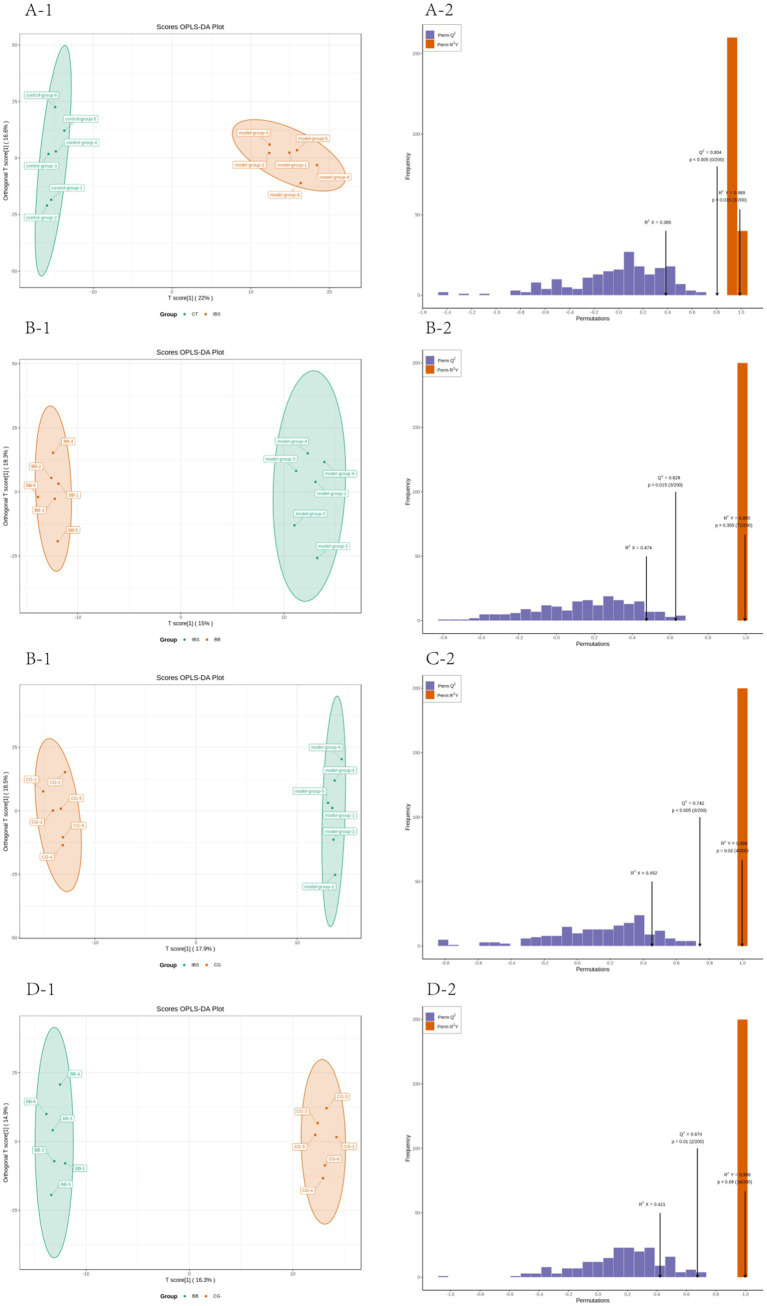
Plot of OPLS-DA scores and model evaluation in four groups of rats. **(A1,A2)** *R*^2^*X* = 0.385, *R*^2^*Y* = 0.989, *Q*^2^ = 0.804, *Q*^2^ > 0.5. **(B1,B2)** *R*^2^*X* = 0.474, *R*^2^*Y* = 0.995, *Q*^2^ = 0.629, *Q*^2^ > 0.5. **(C1,C2)** *R*^2^*X* = 0.452, *R*^2^*Y* = 0.998, *Q*^2^ = 0.742, *Q*^2^ > 0.5. **(D1,D2)** *R*^2^*X* = 0.421, *R*^2^*Y* = 0.998, *Q*^2^ = 0.674, *Q*^2^ > 0.5.

Orthogonal partial least squares discriminant analysis (OPLS-DA) combines orthogonal signal correction (OSC) and PLS-DA methods, and is able to decompose the X matrix information into two types of information related to Y and irrelevant information, and screen the discrepant variables by removing the irrelevant differences. The metabolome data were analyzed according to the OPLS-DA model, and the irrelevant differences were removed to screen the difference variables and evaluate the stability of the model. The results demonstrated significant separation between the IBS group and the CT group, the IBS group and the BB group, as well as the IBS group and the CG group. Additionally, significant separation was observed between the BB group and the CG group (Figure 6).

### 3.7 Differential metabolite screening

The measured metabolites were compared across the four sample groups using PCA and OPLS-DA, followed by univariate statistical analysis to compare metabolites between each pair of groups. Metabolites with fold change ≥1.5 and fold change ≤0.67 and VIP ≥1 were selected as significant differences. The results showed that, compared to the CT group, the IBS group exhibited 249 differential metabolites, including 103 down-regulated and 146 up-regulated. In the BB group compared to the IBS group, 122 differential metabolites were identified, with 88 down-regulated and 34 up-regulated. There were 160 differential metabolites in the CG group compared with the IBS group, 94 down-regulated and 66 up-regulated; 119 differential metabolites in the BB group compared with the CG group, 40 down-regulated and 79 up-regulated. Some of the results of the differential metabolite screening are shown in Table 1.

**Table 1:** Selected differential metabolite screening tables.

	Number	Metabolite name	Fold change	VIP	Trend
CT vs. IBS	1	Adenosine diphosphate (adenosine 5′-diphosphate)	10.917123	1.726568	Up
	2	Thromboxane B2 (TXB2)	1.590376	1.125527	Up
	3	Malonic acid (malonic acid)	0.365072	1.458834	Down
	4	L-Leucyl-L-glycine (Leu-Gly)	0.491794	1.425684	Down
	5	Palmitoleic acid (FFA(16:1))	0.488608	1.797867	Down
	6	Prostaglandin E2	2.055582	1.356133	Up
	7	Prostaglandin F2α (PGF2α)	2.570508	1.602108	Up
	9	Prostaglandin J2 (PGJ2)	614.259259	1.084081	Up
	10	L-Phenylalanyl-L-asparagine (Phe-Asn)	0.001112	1.272098	Down
	11	5-Hydroxyindole-3-acetic acid	0.435882	1.486065	Down
	12	(R)-3-Hydroxybutanoic acid	0.368104	1.478544	Down
IBS vs. BB	1	Adenosine 5′-monophosphate	0.581436	2.127294	Down
	2	γ-L-Glutamate-cysteine	0.454975	1.455504	Down
	3	Adenosine 5′-diphosphate	0.027199	2.419959	Down
	4	Glycyl-L-proline (Glyc-Pro)	0.136144	1.771296	Down
	5	15S-Hydroxy-8Z,11Z,13E-octadecatrienoic acid (15(S)-HETrE)	4.746481	1.611142	Up
	6	Urobilin	432.1	1.833054	Up
	7	N-Acetyl-D-galactosamine	0.061003	1.013921	Down
	8	γ-Aminobutyric acid	1.62677	1.914534	Up
	9	N4-Acetylcytidine	0.482979	2.372582	Down
	10	Kynurenine	0.583675	1.571724	Down
IBS vs. CG	1	Thromboxane B2 (XB2)	0.389020	1.853065	Down
	2	1-Methyluric acid	0.488279	1.669248	Down
	3	Prostaglandin E2	0.449367	1.551774	Down
	4	Prostaglandin F2α (PGF2α)	0.432796	1.587383	Down
	5	Prostaglandin J2 (PGJ2)	0.001623	1.035045	Down
	6	Prostaglandin F1α (6 keto-PGF1α)	0.147452	1.736029	Down
	7	Uric acid	0.341566	1.632529	Down
	8	N4-Acetylcytidine	0.490369	2.046989	Down
	9	Isoleucine-methionine (Ile-Met)	3.452513	1.223360	Up
	10	2,4-Dihydroxypyridine	0.497661	1.572418	Down
BB vs. CG	1	L-Cystathionine	2.909432	1.247029	Up
	2	Thromboxane B2 (TXB2)	0.484587	1.790735	Down
	3	Deoxyguanosine diphosphate (dGDP)	36.863202	2.057254	Up
	4	Prostaglandin J2 (PGJ2)	0.001521	1.423545	Down
	5	Tryptophanyl-glutamate (Trp-Glu)	2.134898	1.801048	Up
	6	15S-hydroxy-8Z,11Z,13E-octadecatrienoic acid (15(S)-HETrE)	0.165558	1.659601	Down
	7	Glycyl-L-proline (Glyc-Pro)	7.439536	1.155539	Up
	8	L-Leucyl-L-valine (Leu-Val)	2.037116	1.193786	Up
	9	Threonyl-val-leucine (Thr-Val-Leu)	2.530749	1.049783	Up
	10	Isoleucine-methionine (Ile-Met)	3.859779	1.594698	Up
	11	Farnesylacetone	2.522340	1.438857	Up

**Figure 7:**
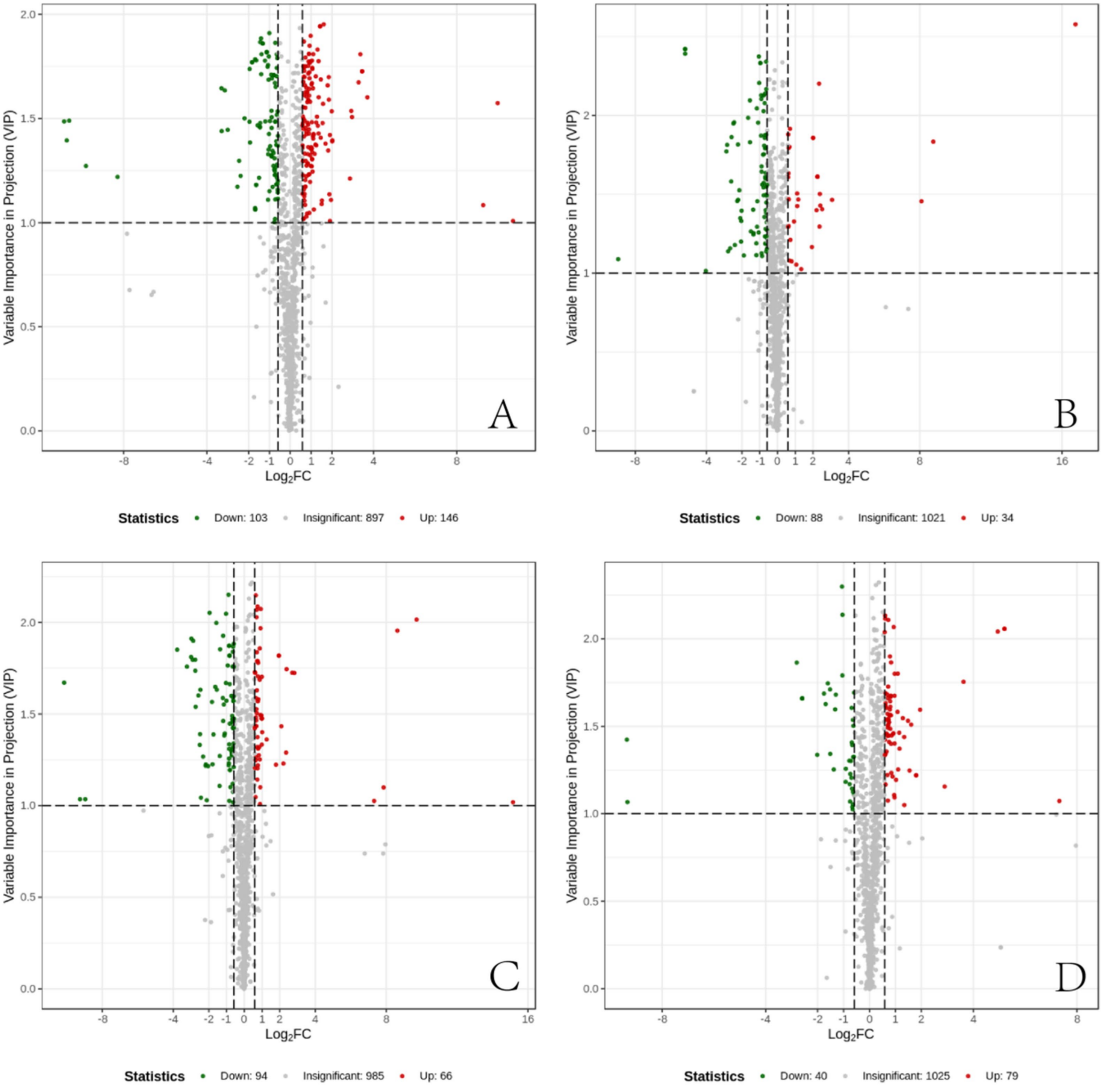
Differential metabolite volcano plots. **(A)** Differential metabolites between the CT group and the IBS group. **(B)** Differential metabolites between the IBS group and the BB group. **(C)** Differential metabolites between the CG group and the IBS group. **(D)** Differential metabolites between the BB group and the CG group. Each point in the volcano plot represents a metabolite. The horizontal axis represents the log2 fold change of metabolite quantification between two samples, while the vertical axis represents the VIP value. A larger absolute value on the horizontal axis indicates more pronounced differential expression in terms of fold change, whereas a larger value on the vertical axis indicates greater statistical significance and robustness of the differentially expressed metabolites. Green points represent down-regulated metabolites, red points represent up-regulated metabolites, and black points represent metabolites with no significant differences.

The differential metabolites between each pair of sample groups were visualized using volcano plots (Figure 7). These plots demonstrate the differences in metabolite expression levels and their statistical significance between each pair of groups.

**Figure 8:**
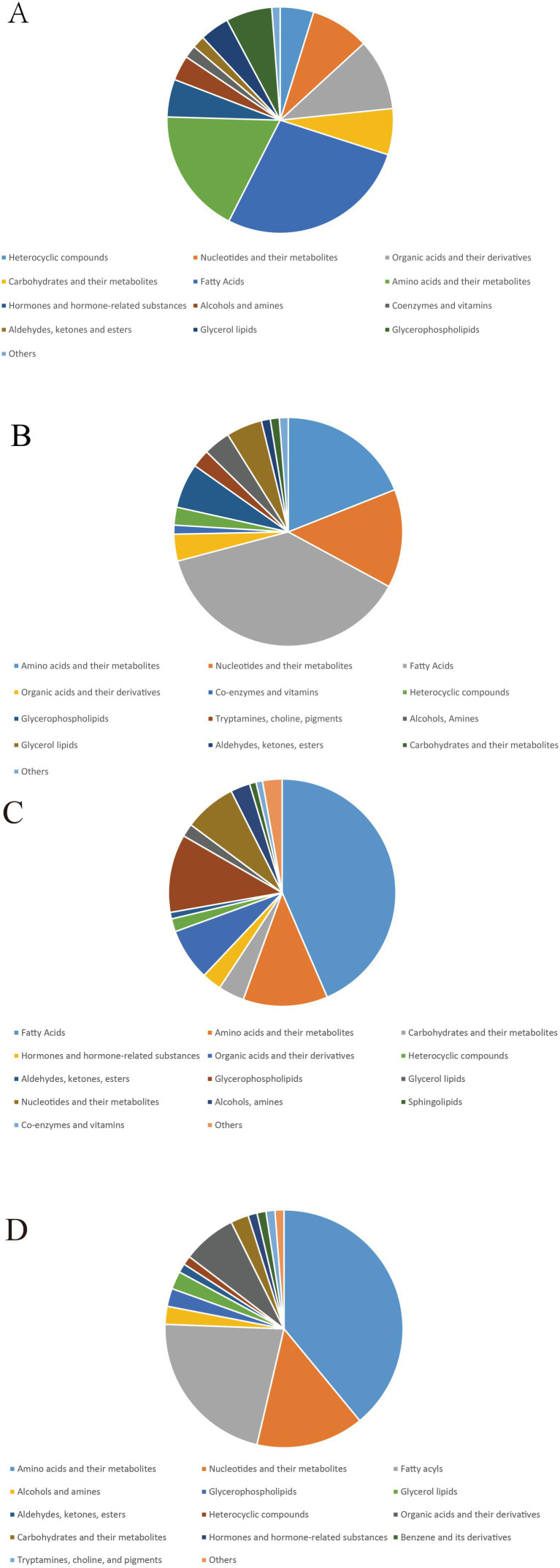
Pie chart of the percentage of categorization of different metabolite substances. **(A)** CT group compared with IBS-D group. **(B)** IBS-D group compared with BB group. **(C)** IBS group compared with CG group. **(D)** BB group compared with CG group.

Substance categorization of differential metabolites and presentation of percentages through pie charts (Figure 8). Compared to the CT group, the IBS group showed that fatty acyls were the most prevalent category of differential metabolites, followed by amino acids and their metabolites, hormones and hormone-related substances, glycerophospholipids, and nucleotides and their metabolites. The BB group exhibited fatty acyls as the most abundant category compared to the IBS group, followed by amino acids and their metabolites, hormones and hormone-related substances, and organic acids and their derivatives. The CG group demonstrated that fatty acyls accounted for the largest proportion of differential metabolites compared to the IBS group, followed by bile acids, amino acids and their metabolites, carbohydrates and their metabolites, and organic acids and their derivatives. The BB group showed that bile acids represented the largest proportion of differential metabolites compared to the CG group, followed by amino acids and their metabolites, carbohydrates and their metabolites, and benzene and its derivatives.

### 3.8 Screening for identical differential metabolites with different trend

**Figure 9:**
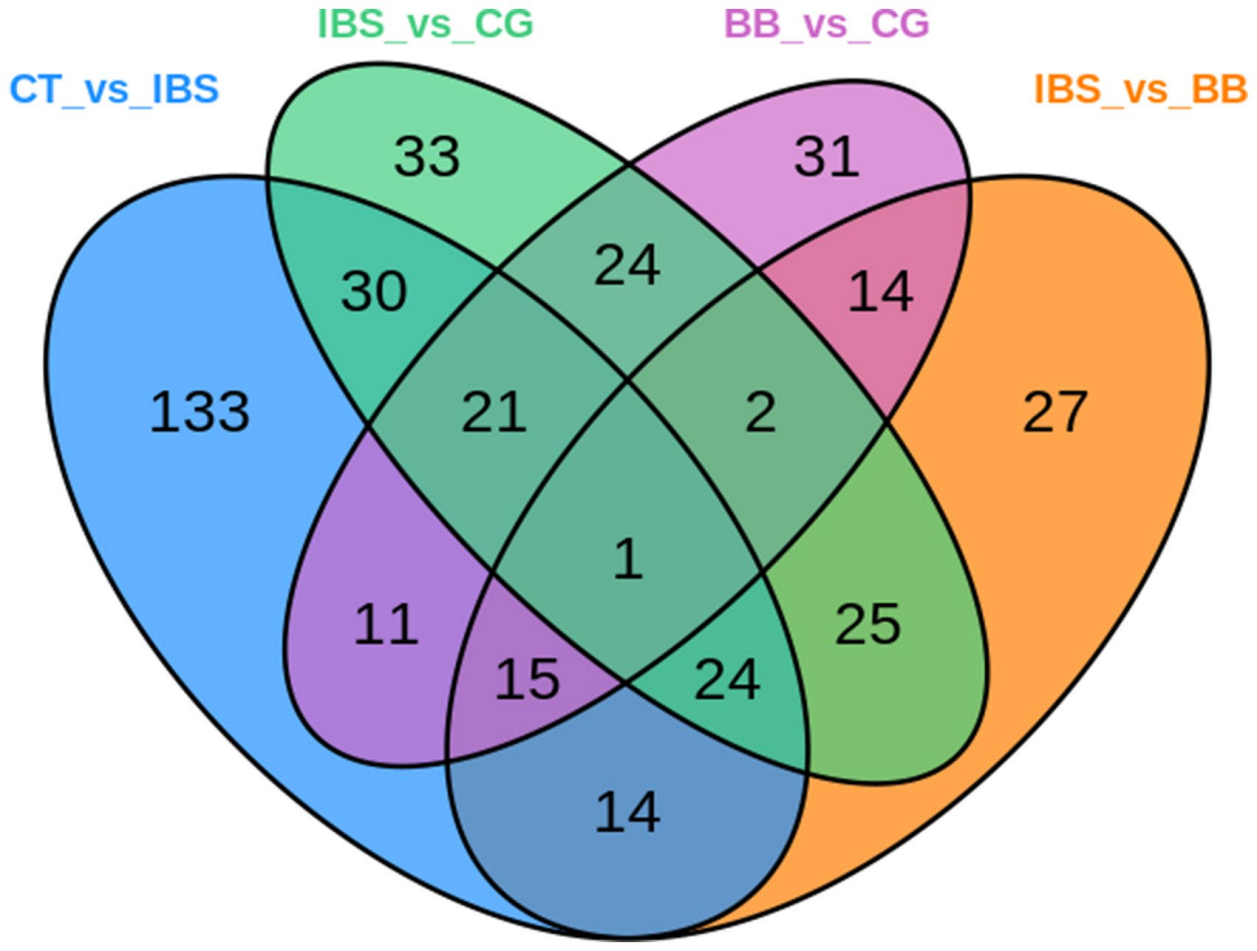
Venn diagrams of differential metabolites by group. Each circle represents a comparison group. Overlapping regions indicate the number of differential metabolites shared between groups, while non-overlapping regions indicate the number of differential metabolites unique to each group.

The relationship between the differential metabolites in each group was visualized using a Venn diagram (Figure 9). The results showed that 54 differential metabolites were common to the comparisons between the CT group and the IBS group and between the IBS group and the BB group, while 76 were shared between the comparisons between the CT group and the IBS group and between the IBS group and the CG group. Additionally, 25 differential metabolites were consistently identified across the comparisons between the CT group and the IBS group, between the IBS group and the BB group, and between the IBS group and the CG group. Only one identical differential metabolite was found across all four comparisons.

### 3.9 Metabolic pathway enrichment analysis

**Figure 10:**
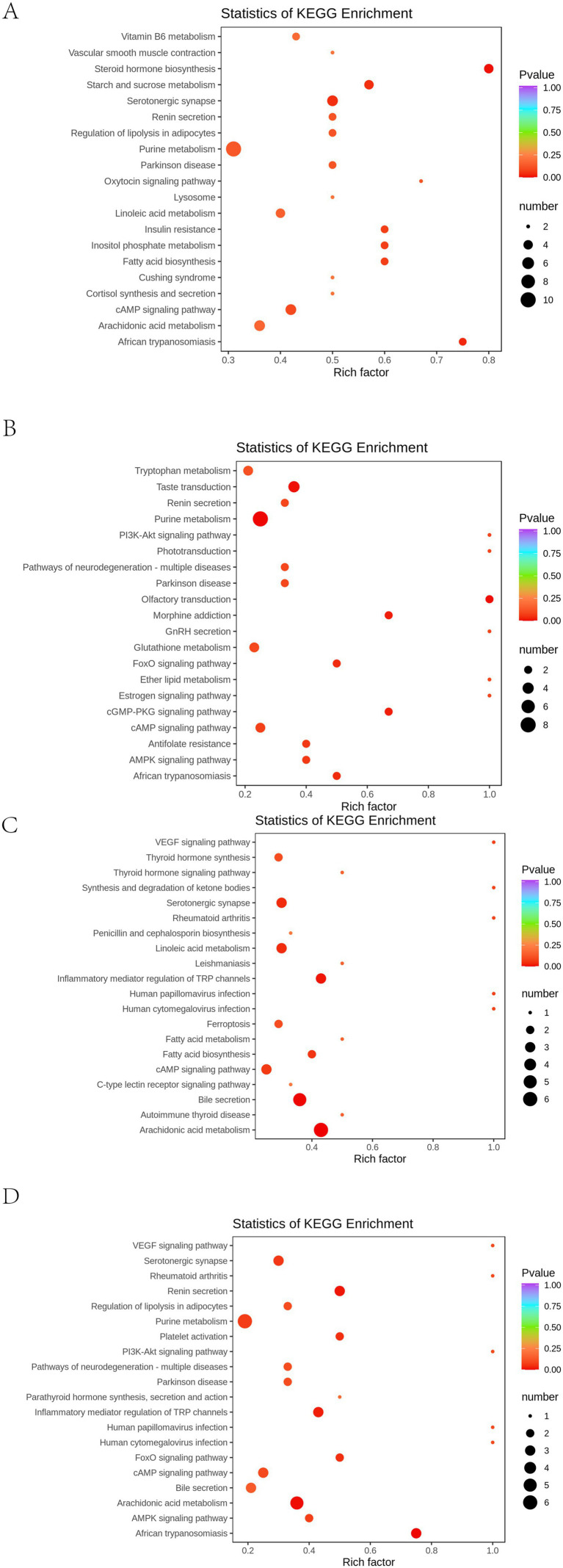
KEGG enrichment maps of differential metabolites across groups. **(A)** CT group compared with IBS group. **(B)** IBS group compared with BB group. **(C)** IBS group compared with CG group. **(D)** BB group compared with CG group. The horizontal axis indicates the corresponding Rich factor for each pathway. The larger the ratio, the greater the enrichment. Vertical axis are pathway names. The point color indicates the *p*-value, with redder colors representing more significant enrichment. The point size corresponds to the number of enriched differential metabolites.

The differential metabolites were systematically categorized and annotated based on the diverse biochemical pathways outlined in the KEGG (Kyoto Encyclopedia of Genes and Genomes) database. These metabolites were further classified according to the specific pathways they participate in, followed by enrichment analysis (Figure 10). Compared to the CT group, the IBS group showed changes in 112 metabolic pathways, of which 4 demonstrated statistically significant differences (*p* < 0.05): steroid hormone biosynthesis, African trypanosomiasis, starch and sucrose metabolism, and the 5-hydroxytryptamine metabolic pathway (Figure 10A). Compared to the IBS group, the BB group exhibited differences in 74 metabolic pathways, with seven pathways reaching statistical significance (*p* < 0.05): purine metabolism, olfactory transduction, phototransduction, cGMP-PKG signaling, African trypanosomiasis, FoxO signaling, and the morphine addiction pathway (Figure 10B). Compared to the IBS group, the CG group displayed variations in 64 metabolic pathways, among which five were statistically significant (*p* < 0.05): arachidonic acid metabolism, bile secretion, 5-hydroxytryptamine metabolism, inflammatory mediator regulation of TRP channels, and linoleic acid metabolism (Figure 10C). Furthermore, comparison between the BB group and the CG group revealed differences in 71 metabolic pathways, with six pathways showing statistical significance (*p* < 0.05): arachidonic acid metabolism, African trypanosomiasis, renin secretion, inflammatory mediator regulation of TRP channels, FoxO signaling pathway, and platelet activation (Figure 10D).

### 3.10 Identification of key differential metabolites

Differential metabolites were identified for each group comparison based on fold change ≥1.5 and fold change ≤0.67 and VIP ≥1. Metabolic pathway analysis using the KEGG database revealed significant differences in several pathways (*p* < 0.05). Further analysis showed that some differential metabolites exhibited distinct trends across comparison groups, as depicted in Venn diagrams, and were associated with specific KEGG pathways. These metabolites were identified as key metabolites in this study. Detailed results are shown in Table 2.

**Table 2:** Identical differential metabolites across comparison groups.

Substance	Chemical formula	Trend				Metabolic pathway
		CT vs. IBS	IBS vs. BB	IBS vs. CG	BB vs. CG	
Adenosine 5′-diphosphate	C_10_H_15_N_5_O_10_P_2_	Up	Down	—	Up	OxPhos; Purine metab.; Cofactor biosynth.; FoxO; Neuroactive L-R; Lysosomes; AMPK; Platelet activ.; Thermogenesis; Taste transduct.; Renin secret.
TXB_2_	C_20_H_34_O_6_	Up	—	Down	Down	Arachidonic acid metab.; 5-HT synapse; Bile secretion.
(±)15-HETE	C_20_H_32_O_3_	—	—	Down	Down	Arachidonic acid metab.; TRP channel inflam.mediator reg.
Prostaglandin E2	C_20_H_32_O_5_	Up	—	Down	—	Arachidonic acid metab.; cAMP signaling pathway; Neuroactive ligand-receptor interact.; C-type lectin receptor signaling; 5-HT synapse; TRP channel inflam. Mediator reg.; Oxytocin signaling pathway; Adipocyte lipolysis reg.; Renin secretion;
PGJ2	C_20_H_30_O_4_	Up	—	Down	Down	Arachidonic acid metab.; 5-HT.
2-Deoxyribose 1-phosphate	C_5_H_11_O_7_P	Up	Down	Down	Down	Pentose phosphate pathway; Pyrimidine metab.
Deoxyguanosine diphosphate (dGDP)	C_10_H_15_N_5_O_10_P_2_	Up	Down	—	Up	Purine metab.
5′-Adenylyl sulfate (APS)	C_10_H_14_N_5_O_10_PS	Up	Down	—	Up	Purine metab.; Monobactam biosynth.; Sulfur metab.
N-acetylornithine	C_7_H_14_N_2_O_3_	Down	Up	Up	—	Arginine biosynth.; 2-Oxocarboxylic acid metab.; Amino acid biosynth.
Inosine diphosphate	C_10_H_14_N_4_O_11_P_2_	Down	Down	—	—	Purine metab.
2-Deoxyribose-5′-phosphate	C_5_H_11_O_7_P	Up	Down	—	—	Pentose phosphate pathway.
Kinurenine	C_10_H_12_N_2_O_3_	Up	Down	—	Up	Tryptophan metab.; Cofactor biosynth.; African trypanosomiasis.
Kynurenic acid	C_10_H_7_NO_3_	Down	Down	—	—	Tryptophan metab.

## 4 Discussion

The heart - gut interactions in IBS are co-mediated through dual neural and humoral pathways. Firstly, the NTS in the medulla oblongata serves as a common integration center for cardiac and intestinal neural signals. Vagal and spinal primary afferent nerve endings form specialized receptors in the submucosal and myenteric plexuses of the intestinal wall (40), which can sensitively detect abnormal mechanical distension, chemical stimuli, and inflammatory mediators in the gut (41). These abnormal signals are then transmitted upward by afferent nerves and converge at the NTS (13). Here, visceral signals are integrated and processed, and through “crosstalk” between different neuronal populations within the NTS, ultimately interfere with cardiac autonomic innervation (17). Increased sympathetic nervous activity acts on β-adrenergic receptors in cardiomyocytes through norepinephrine release, altering myocardial energy metabolism preference by promoting glycolysis and fatty acid oxidation, while simultaneously increasing heart rate and myocardial oxygen consumption (42). Decreased vagal tone impairs its homeostatic regulatory capacity on cardiac metabolic activity, reduces acetylcholine release and its direct inhibitory effects on myocardial metabolic pathways, thereby disrupting fine cardiac regulation (43). This dual change in autonomic nervous output constitutes the neural pathway connecting intestinal pathology with cardiac metabolic changes.

Meanwhile, the systemic humoral pathway closely interacts with the neural pathway, collectively amplifying the heart-gut interplay. The initiation of the humoral pathway originates within the intestinal tract itself. In the context of IBS, the function of the intestinal epithelial barrier is impaired, resulting in a marked increase in intestinal permeability (44, 45). This alteration facilitates the access of gut microbes and their products, such as LPS, to the mucosal immune system and promotes their activation of immune pathways (46, 47). Consequently, local innate and adaptive immune responses are triggered, which include the activation of mast cells and the release of pro-inflammatory cytokines (48–50). This aberrant immune activation may further extend into the systemic circulation, leading to a quantifiable low-grade inflammatory state (51–53). In clinical studies, elevated markers such as the systemic immune-inflammation index (SII) in peripheral blood provide evidence for this phenomenon (54). Circulating inflammatory mediators, including TNF-α and IL-6, can directly act on the cardiac microvascular endothelium to promote local inflammation (55). Moreover, these mediators are capable of crossing the blood–brain barrier or modulating central autonomic nuclei via regional signaling, thereby exacerbating pre-existing autonomic dysregulation at the humoral level (56). Ultimately, this gut-originated, humorally disseminated inflammatory signaling pathway acts in concert with the aforementioned neural mechanisms, collectively contributing to manifestations of cardiac autonomic dysfunction, such as reduced HRV.

The modeling method employed in our experiment has been demonstrated to effectively simulate key pathophysiological features of IBS, including reduced sucrose preference, increased fecal output, enhanced visceral pain response to colorectal distension, and altered physiological functions of the colonic mucosa in rats subjected to CAS (39). Negative emotions can activate the hypothalamic–pituitary–adrenal axis (HPA), leading to increased sympathetic nerve activity, parasympathetic nerve inhibition, and a decrease in the overall regulatory ability and adaptability of the ANS. The main manifestations include a decrease in SDNN, a significant decrease in RMSSD and HF, and an increase in the LF/HF ratio (17). Enhanced sympathetic nerve activity directly increases intestinal smooth muscle contraction and visceral pain conduction by releasing norepinephrine, resulting in abdominal pain and abnormal defecation (57). Vagal nerve hypofunction can delay colonic transit (IBS-C) or reduce intestinal mucosal barrier protection (IBS-D), further exacerbating symptoms (58, 59). Functional MRI studies have confirmed that in patients with IBS, when the colon is dilated, abnormal HRV is associated with overactivation of brain pain management territories such as the insula and anterior cingulate gyrus (60). This further illustrates that the visceral hypersensitivity of IBS originates from the interaction between ANS dysfunction and overactivation of the central pain management system. Consistent with previous studies, in this study, the visceral pain threshold of IBS rats modeled by CAS decreased. Meanwhile, HRV indicators reflected an imbalance of the autonomic nervous system. Previous studies have shown that acupuncture can effectively regulate ANS function, such as stimulating PC6 can enhance vagal activity (33), improve sympathetic-vagal balance under fatigue state (34), and moxibustion at CV4 and ST36 can also regulate heart rate variability with a sustained effect (31). The results of this study showed that acupuncture with the combination of primary and secondary acupoints significantly improved HF and LF/HF in IBS rats, suggesting that it may regulate HRV by restoring ANS balance.

As members of the natriuretic peptide family, ANP and BNP are classically thought to be driven by atrial stretch or ventricular mechanical load, respectively, and together regulate water-salt balance and cardiovascular homeostasis. However, existing evidence suggests that ANP release is not only regulated by preload but can also occur independently of changes in central Volume blood. Exogenous administration of Epinephrine can significantly increase serum MR-proANP levels, suggesting that catecholamines may directly or indirectly promote ANP release, and increased sympathetic nerve activity may be one of its regulatory pathways (61). In addition, studies have shown that ANP may be involved in the negative regulation of the HPA axis by inhibiting the release of CRH and ACTH in the central nervous system (62). However, the increased ANP levels in rats with acute and chronic stress observed in this experiment do not contradict the overactivation of their HPA axis. Some studies suggest that this reflects an endogenous compensatory mechanism, which alleviates the physiological load caused by stress through its diuretic, hypotensive, and central anti-stress effects (63). The elevated BNP level is also closely related to the increased sympathetic nerve activity (64), and the sympathetic nerve may indirectly promote its secretion by increasing the cardiac load (65). Combined with the results of heart rate variability in this study, the increases in ANP and BNP are closely related to the overactivation of the sympathetic nervous system under the stress state of IBS, but the specific mechanism needs further study. The BB group can better regulate the levels of ANP and BNP. To further explore the intracellular mechanisms in the heart of IBS rats, we employed cardiac metabolomics analysis.

Adenosine diphosphate (ADP) and adenosine monophosphate (AMP) are core members of the cell energy metabolism, and together with adenosine triphosphate (ATP), they form the cell’s adenylate pool (66). During the process of accelerated energy consumption, ATP is hydrolyzed in large quantities, directly leading to a significant increase in the concentration of its direct product ADP (67, 68). Subsequently, the accumulated ADP activates adenylate kinase (AK) to catalyze the reaction 2 ADP → ATP + AMP (69, 70), buffering the consumption of its storage capacity through regenerating ATP and maintaining the stability of ATP concentration (71). Meanwhile, the AMP produced by this reaction should have accumulated, but its concentration remains unchanged, indicating that it is rapidly used to activate AMP-activated protein kinase (AMPK) (72). This indicates that the energy stress signal has been activated and is continuously operating (73). Therefore, the increase in ADP and the dynamic stability of combined ATP and AMP in the model group indicate that the heart is in an energy stress state with high metabolic flux. This metabolic state may be associated with increased cardiac load and decreased efficiency, which could be linked to the observed continuous excitation of the sympathetic nerve in the IBS model. Biao-Ben acupoint combination may effectively reduce unnecessary energy load and consumption of the heart, an effect that coincides with the observed regulation of autonomic nerve balance. The observed reduction in ADP levels suggests a potential decrease in the rate of ATP hydrolysis, indicating an improvement in cardiac energy expenditure. The decrease in ADP was accompanied by a reduction in AMP, which is consistent with a downstream weakening of the adenylate kinase (AK) reaction. Therefore, the simultaneous decrease in ADP and AMP is consistent with an improvement in the cardiac “high metabolic flux, low efficiency” stress state induced by the IBS model.

In addition, as a common endogenous ligand for multiple subtypes such as P2Y1R and P2Y12R, ADP regulates calcium mobilization, neural excitability, and immunoinflammatory reactions in the local microenvironment (74–76). P2Y receptors are key molecules mediating visceral pain. The P2Y1 receptor is highly expressed in the colonic submucosal plexus and directly participates in mediating visceral hypersensitivity signals (77, 78). The P2Y12 receptor drives neuroinflammation and chronic hyperalgesia by activating spinal microglia (79). Notably, the regulation of P2Y receptors by acupuncture has become an effective strategy for relieving pain. Electroacupuncture can improve inflammatory pain by inhibiting the expression of P2Y12 receptors in the spinal dorsal horn and reducing microglial M1 polarization (80). It can also alleviate IBS visceral hypersensitivity by blocking the P2Y1/MAPK/ERK pathway and inhibiting astrocyte activation (81). Moxibustion can alleviate macrophage inflammatory reaction by inhibiting the P2Y12/PI3K/AKT pathway (82). In addition, electroacupuncture can improve pain-emotion comorbidity by regulating the prefrontal P2Y12 receptor or the P2Y1 receptor in social stress models (83, 84).

Therefore, we hypothesize that the therapeutic effect of the Biao-Ben acupoint combination on IBS constitutes a multi-system synergistic effect, characterized by concurrent improvements in cardiac energy metabolism and visceral pain threshold. As supported by previous literature, these parallel improvements likely arise from interconnected mechanisms, potentially involving the modulation of P2Y receptor signaling pathways at different levels. The synchronous improvement in cardiac metabolic function and visceral sensitivity further suggests that acupuncture may produce coordinated therapeutic effects across multiple organ systems, potentially associated with the regulation of the autonomic nervous system.

In addition to energy metabolic disorder, the inflammatory stress state of the heart is another important finding. The kynurenine pathway is the main pathway of tryptophan metabolism, accounting for approximately 95% of the metabolic flux (85). This pathway is initiated by the rate-limiting enzyme tryptophan-2,3-dioxygenase or indoleamine-2,3-dioxygenase (IDO) (86). It is associated with immune dysfunction, Disorder central nervous system, autoimmunity, and Infection (87). IDO1 promotes the conversion of tryptophan to kynurenine (KYN), which can be further metabolized into neurotic toxicity quinolinic acid or neuroprotective kynurenic acid (KYNA) (88). We observed that the contents of KYN and KYNA in the heart of the IBS model group were significantly increased, while the levels of the substrate tryptophan and the bypass product precursor serotonin did not change significantly. It indicates that IDO1, the rate-limiting enzyme of KYN, is specifically activated. As mentioned earlier, within the systemic inflammatory state triggered by IBS-induced intestinal barrier damage, circulating inflammatory mediators (such as IFN-*γ*) can act on peripheral organs including the heart, potently inducing the expression of IDO1. These circulating inflammatory mediators such as IFN-γ can act on peripheral organs such as the heart, strongly induce the expression of IDO1 (89, 90), which is associated with a shift in local cardiac tryptophan metabolism towards the kynurenine pathway and the subsequent accumulation of KYN and its metabolites in heart tissue. Previous studies have confirmed that acupuncture and moxibustion can effectively inhibit the release of pro-inflammatory cytokines (TNF-*α*, IL-6, IFN-γ) in rats with IBS and IBD (91, 92), and enhance intestinal barrier function and reduce intestinal permeability by up-regulating the expression of aquaporins (AQPs) and tight junction proteins (ZO-1, occludin) (93). Based on this, we speculate that the down-regulating effect of acupuncture with the combination of acupoints for both the root cause and symptoms on cardiac kynurenine and kynurenic acid may indirectly result from its systematic regulation of IBS intestinal inflammation.

This study has several limitations. First, the research did not include mechanistic validation such as nerve blockade or receptor pathway verification. Second, although *a priori* statistical power analysis was conducted to estimate the sample size, the use of only six subjects per group may limit the generalizability of the metabolomic variability analysis. Third, while the CAS modeling approach employed is a widely accepted method for establishing IBS models, it may introduce confounding effects that independently influence HRV and cardiac metabolism. Future studies should consider utilizing different modeling strategies, such as chronic water avoidance stress, trinitrobenzene sulfonic acid enema, or chemical stimulation combined with stress models. Finally, during HRV measurements, light isoflurane anesthesia was administered to avoid restraint stress in rats. Although this ensured that all animals were assessed under highly consistent physiological conditions and reduced intergroup variability, it must be acknowledged that the anesthetic agent itself affects HRV recordings.

## 5 Conclusion

In conclusion, Biao-Ben acupoint combination significantly improves HRV in IBS rats by regulating the balance of the autonomic nervous system, while also regulating the levels of cardiac neuroendocrine markers ANP and BNP. Cardiac metabolomics analysis shows that this therapy can reduce the contents of ADP and AMP, improve the state of cardiac energy metabolism, and decrease the levels of kynurenine pathway metabolites KYN and KYNA. This study holds clear clinical value. Biao-Ben acupoint combination can serve as a complementary therapy, particularly suitable for IBS patients with reduced HRV, as it can comprehensively improve their cardio-intestinal symptoms. Furthermore, BB demonstrates significantly superior efficacy over CG in regulating cardiac metabolism and HRV, suggesting that acupoint combinations should be based on specific biological foundations. Therefore, this study provides an important scientific basis for optimizing clinical acupuncture point selection protocols.

## Data Availability

The datasets presented in this study can be found in online repositories. The names of the repository/repositories and accession number(s) can be found in the article/supplementary material.

## Footnotes

1
http://www.hmdb.ca

2
https://mona.fiehnlab.ucdavis.edu

3
https://massbank.jp

4
https://metlin.scripps.edu

5
https://www.nist.gov

6
http://metdna.zhulab.cn

7
https://www.kegg.jp
